# Evaluation of Blue Crab, *Callinectes sapidus*, Megalopal Settlement and Condition during the Deepwater Horizon Oil Spill

**DOI:** 10.1371/journal.pone.0135791

**Published:** 2015-08-13

**Authors:** Erin K. Grey, Susan C. Chiasson, Hannah G. Williams, Victoria J. Troeger, Caz M. Taylor

**Affiliations:** 1 Division of Chemistry and Biological Sciences, Governors State University, University Park, Illinois, United States of America; 2 Department of Ecology and Evolutionary Biology, Tulane University, New Orleans, Louisiana, United States of America; 3 College of Veterinary Medicine, University of Florida, Gainesville, Florida, United States of America; 4 Rollins School of Public Health, Emory University, Atlanta, Georgia, United States of America; Department of Agriculture, AUSTRALIA

## Abstract

The Blue Crab, *Callinectes sapidus*, is a commercially, culturally, and ecologically significant species in the Gulf of Mexico (GOM), whose offshore stages were likely impacted by the Deepwater Horizon oil spill (DWH). To test for DWH effects and to better understand the planktonic ecology of this species, we monitored *Callinectes spp*. megalopal settlement and condition at sites within and outside of the spill extent during and one year after the DWH. We tested for DWH effects by comparing 2010 settlement against baseline data available for two sites, and by testing for differences in settlement and condition inside and outside of the spill extent. We also developed time series models to better understand natural drivers of daily settlement variation (seasonal and lunar trends, hydrodynamics, wind) during 2010 and 2011. Overall, we found that neither megalopal settlement nor body weight were significantly reduced at oiled sites, but that high unexplained variation and low statistical power made detection of even large effects unlikely. Time series models revealed remarkably consistent and relatively strong seasonal and lunar trends within sites (explaining on average 28% and 9% of variation, respectively), while wind and hydrodynamic effects were weak (1–5% variation explained) and variable among sites. This study provides insights into DWH impacts as well as the natural drivers of *Callinectes spp*. megalopal settlement across the northern GOM.

## Introduction

Understanding the causes of natural variation in ecological dynamics is important for establishing baselines against which to detect significant perturbations. The Deepwater Horizon oil spill (DWH), for example, likely impacted the offshore stages of the Blue Crab, *Callinectes sapidus*. *C*. *sapidus* is abundant along the western Atlantic and Gulf of Mexico (GOM) coasts where it supports large commercial and recreational fisheries [1] and is an integral part of estuarine and offshore food webs. Like many coastal species, *C*. *sapidus* has a complex, spatially-structured life history where adults reside in estuarine habitats and gravid females swim offshore to spawn their eggs in higher salinity waters. The eggs quickly hatch into planktonic, surface-dwelling zoeae, which after one to two months molt into post-larval megalopae [2] that use vertical swimming behaviors to settle back into estuarine habitats [3]. The timing of the DWH spill, in which oil flowed from the well from April 20th–July 15^th^ 2010, coincided with the peak in Blue Crab spawning in the northern GOM [1] and surface oil was present throughout much of the known and suspected spawning areas [1, 4, 5]. Oil and oil-dispersant mixtures are known to have lethal and sub-lethal effects on Blue Crab megalopae and juveniles, such as reduced RNA:DNA, protein:DNA and lipid content [6–9], and oil derived carbon has been shown to have entered the planktonic food web during the DWH [10]. Thus, it is very likely that the offshore *C*. *sapidus* stages were affected by the DWH event.

Documenting DWH effects on *C*. *sapidus* has been challenging because its offshore life history is relatively poorly understood, particularly in the GOM. *C*. *sapidus* megalopal settlement into estuaries is known to be extremely variable both among sites and within the same sites over time [11, 12], but there is little consensus on the causes of this variation. Previous studies have attributed some of this variability to seasonal, lunar, meteorological, or hydrological drivers as outlined in Table 1 [11–25], but have collectively failed to identify consistent drivers across sites or years [26]. Given that the independent variables and statistical methods varied among these studies, the effects of different drivers on *C*. *sapidus* megalopal settlement remain unclear.

**Table 1:** Review of previously-published analyses of Blue Crab megalopae daily settlement rates.

Study	Region (Locale)	Years	Season	Lunar	Auto-correlation	Hydro-dynamics	Wind	Other
*Goodrich *et al*. 1989 [13]	MAB (Chesapeake)	1985–1987	—-	**trend**	—-	**bay volume**	—-	—-
Van Montfrans *et al*. 1990 [14]	MAB (Chesapeake)	1985–1988	—-	**quarter (3rd)**	—-	**sea level flux** ^**a**^	—-	—-
Boylan & Wenner 1993 [15]	SAB (Charleston)	1987–1988	—-	**quarter (4th), days (7, 24)**	—-	**sea level flux**	**speed, direction**	**bottom temp, bottom salinity,** surface temp, surface salinity, precipitation
Jones & Epifanio 1995 [16]	MAB (Delaware)	1989–1992	—-	Quarter	—-	spring tide, **sea level anomaly**	**alongshore speed**	—-
Mense *et al*. 1995 [17]	SAB (North Carolina)	1990–1992	—-	**quarter** ^**b**^ **(1st, 4th)**	—-	—-	speed, **direction**	surface temp, air temp, surface salinity
Perry *et al*. 1995[18]	GOM (Mississippi)	1991–1992	—-	quarters	—-	predicted flux	speed, direction	**temp, salinity**
Van Montfrans *et al*. 1995[11]	MAB (various)	1989–1992	—-	**quarter (variable)**	—-	—-	—-	—-
Rabalais *et al*. 1995 [12] (baseline this study)	GOM (various)	1990–1992	—-	**quarter** ^**b**^ **(1st)** ^**c**^	—-	sea level flux	direction	surface temp, surface salinity
Morgan *et al*. 1996 [19]	GOM (Mobile Bay)	1990–1991	—	Trend	—	sea level flux	**speed, direction**	surface temp
Hasek & Rabalais 2001 [20]	GOM (Louisiana)	1990–1991	—	**quarter** ^**b**^ **(3rd), declination**	—	sea level flux, **sea level max**	speed, direction	surface temp, surface salinity
Spitzer *et al*. 2003 [21]	GOM (Mobile Bay)	1997–1998	—	Quarter	—	sea level flux	**speed, direction,** peak wind	temp, salinity
*Forward *et al*. 2004 [22]	SAB (North Carolina)	1993–2002	**trend**	**Trend**	**3 days**	**night max level**	speed, direction	—
Bishop *et al*. 2010 [23]	SAB (Georgia)	2005	**trend**	—	—	sea level max, **night max level**	**speed, direction**	**temp, surface salinity**
*Eggleston *et al*. 2010 [24]	SAB (Pamlico Sound)	1996–2005	—-	Quarter	**2–3 days**	sea level flux, night flood tide	**speed, direction** ^d^	**storm days**
*Ogburn *et al*. 2012 [25]	SAB (Newport River)	1993–2009	**trend**	quarter ^**b**^	—-	**night flood tides,** tidal range	**speed+ direction**	—
Grey *et al*. 2015 (this study)	GOM (various)	2011–2012	**trend**	**trend**	**yes**	sea level flux, sea level max	alongshore, speed, direction	—-

Regions were defined as MAB = Mid-Atlantic Bight, SAB = South Atlantic Bight, GOM = Gulf of Mexico. Explanatory variables were grouped into six categories: Season = seasonal trends, Lunar = lunar trends or quarters, Autocorrelation = autoregressive process in days, Hydrodynamics = various sea level and tidal metrics, Wind = wind speed or direction, and Other includes various temperature, salinity and storm metrics. For each study, variables found to be significantly associated with daily megalopal settlement are highlighted in bold.

*Settlement pulse correlates also investigated but not included in this table.

^a^Correlation only significant during full moon periods.

^b^Quarters defined as: 1) lunar days 26–4, 2) lunar days 5–11, 3) lunar days 12–18, 4) lunar days 19–25.

^c^Lunar quarter only significant in some years and some sites.

^d^Significant correlation in most years.

In response to the DWH, we conducted a large-scale *C*. *sapidus* megalopae monitoring study at seven sites ranging from Texas to Florida from May-October 2010 and repeated monitoring at four sites in 2011, two of which (Galveston, Dauphin) had baseline data from 1990–1992 [12]. Our goals were to test for DWH effects on megalopal settlement and condition, and to evaluate the strengths of known or suspected natural drivers of megalopal settlement across the GOM. We hypothesized that the spill would have negative effects on *C*. *sapidus* spawning success or early life stage survival or growth, which would manifest as significantly decreased megalopal settlement rates or lower individual weights at affected sites. This hypothesis makes several simplifying assumptions, including that *C*. *sapidus* came into contact with oil or oil-dispersant mixtures, that this contact resulted in increased mortality or reduced growth, that mortality and growth rates would have otherwise been unchanged during 2010, that the proportion of *C*. *sapidus* relative to its co-occurring congener *C*. *similis* remained constant, that megalopae settled near their spawning area, and that the size of the spawning stock was similar among years.

## Materials and Methods

### Data Collection

**Fig 1:**
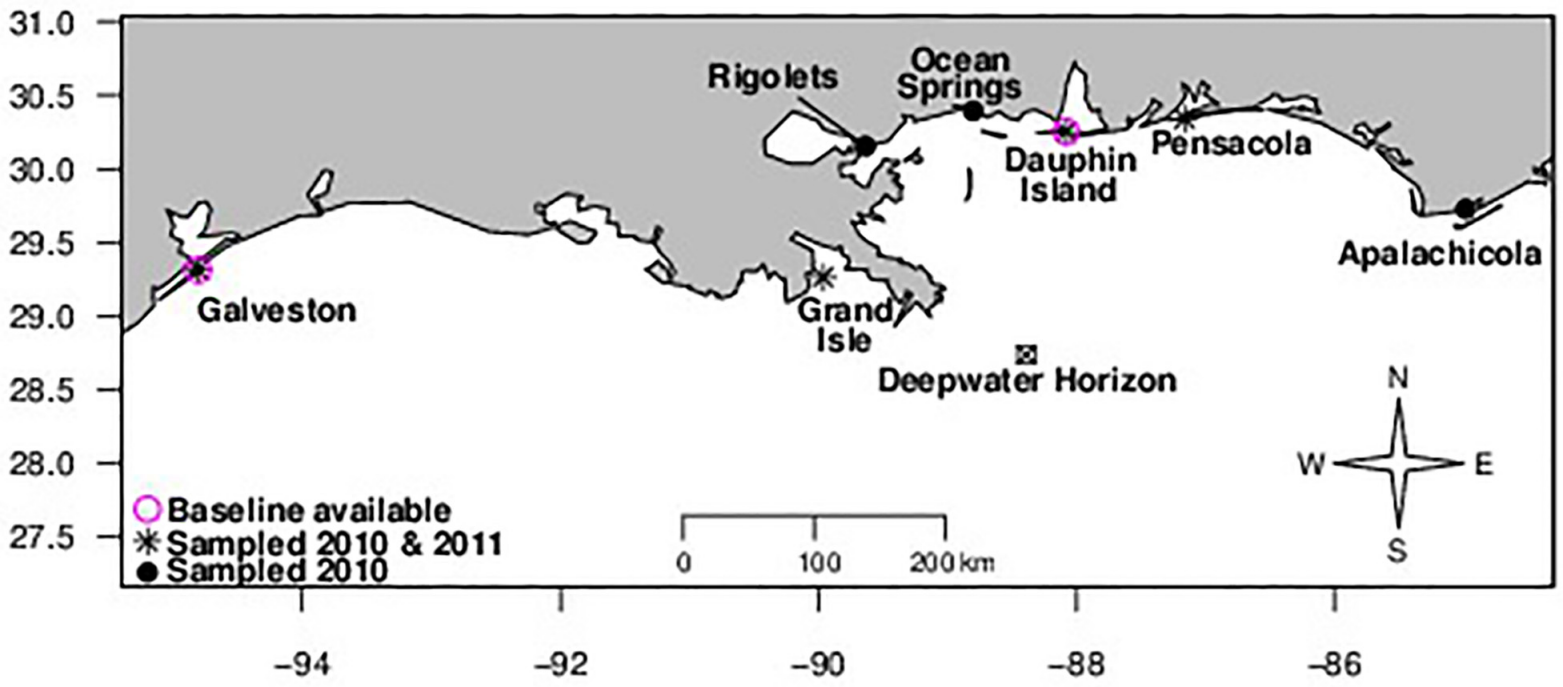
Map of megalopal sampling sites. Sites sampled in 2010 (n = 7) are denoted by a circle, while sites sampled in 2010 and 2011 (n = 4) are denoted by a star. Sites with baseline data from 1990–1992 (Galveston, Dauphin) are marked with a purple circle.

Brachyuran megalopae were collected daily at seven sites in 2010 and four sites in 2011 (Fig 1) using a standard collection method [27] from May or June until October 31^st^. Permission for sampling was obtained from Texas A&M Galveston (Galveston), Sand Dollar Marina (Grand Isle), Fort Pike State Historic Site (Rigolets), Gulf Coast Research Lab (Ocean Springs), Dauphin Island Sea Lab (Dauphin), the Environmental Protection Agency Gulf Ecology Division (Pensacola) and the Apalachicola Maritime Museum (Apalachicola). At each site, for five consecutive days per week, 3–4 hogs hair collectors were deployed in the morning before 10am and then retrieved the following morning, resulting in five 24 hour sampling periods per week. *Callinectes spp*. megalopae were separated from other genera, counted, and stored in ethanol. We attempted identification of *Callinectes* species following Ogburn *et al*. [28]. All were either *C*. *sapidus* or *C*. *similis* but we could not always accurately distinguish the two species and so we refer to our samples as *Callinectes spp*. However, genetic barcoding of megalopae across the sites during August of 2010 found that >95% individuals were *C*. *sapidus* suggesting that *C*. *similis* likely represent a negligible proportion of sampled individuals.

Megalopal condition was measured as mean dry weight for each site from ethanol-fixed samples. To estimate individual weight, we grouped 100 megalopae per site over two-week intervals, including an equal number of individuals from each day when possible. For intervals with <100 collected megalopae, samples of 20 megalopae were used, and intervals with <20 collected megalopae were removed from the analysis. Samples were dried at room temperature in a tissue-covered vial for three days, then dried with nitrogen gas for 15 minutes, and finally dried in a drying oven at 60°C for 30 minutes to obtain dry weight. Samples were chosen randomly for re-weighing to ensure that they were dried to constant mass (+/- 2% mass difference). Samples were weighed on a Sartorius CPA2P balance that was covered to reduce air currents. To ensure that the air humidity had no effect on sample mass, humidity was recorded each day. A representative sample from a more humid day was selected and re-weighed on a less humid day. If the two masses were not within +/-2% of each other, then the samples from the more humid day were further dried and re-weighed. Sample masses were then standardized by dividing the number of megalopae in each sample (either 100 or 20) to attain the mean dry weight per individual for each biweekly period.

Hydrological and meteorological data for time series models were obtained as follows: Daily wind speed (ws) and direction (θ) were downloaded from National Data Buoy Center (http://www.ndbc.noaa.gov/) using the buoy nearest to each site. Only Galveston (buoy #8771510 for 2010 and #8771341 for 2011), Grand Isle (buoy #8761724 both years), Dauphin (buoy #8735180 both years), Pensacola (buoy #8729840 both years) and Apalachicola (buoy #8728690 for 2010) had buoys within 10km. Three wind metrics were calculated for each site from these datasets: the mean daily North-South wind component, [*u* = ws*cos(θ)], the mean daily East-West wind component, [*v* = ws*sin(θ)], and the mean daily alongshore wind component [*a* = ws* cos(θ– x], where x is the nearby coastline’s angle to true North. A positive *u* represents winds blowing to the true north, a positive *v* represents winds blowing to the east, and a positive *a* represents winds blowing northward parallel to the site whereby the Ekman transport process would facilitate water movement landward. Hourly observed sea heights relative to mean sea height (meters) were obtained for each site from nearest NOAA COOPS buoys (http://www.tidesandcurrents.noaa.gov/ofs/ngofs/ngofs.html: Galveston #8771450, Grand Isle #8761724, Dauphin #8735180, Pensacola #8729840, Apalachicola #8728690), from which daily sea height flux (maximum sea height—minimum sea height), maximum daily sea height, and mean sea level at night in meters (averaged over the hours of 8:00pm and 6:00am local time) were calculated. Lunar Day was downloaded from the US Naval Observatory Astronomical Applications Department (http://aa.usno.navy.mil/data/docs/MoonPhase.php). All variables were chosen because they have been investigated in previous Blue Crab settlement studies (Table 1).

### Testing for DWH Effects on Megalopal Settlement and Condition

We tested for DWH effects in two ways. First, at the two sites with previously published daily *C*. *sapidus* settlement data (Galveston, Dauphin) [12], we compared pre-spill settlement rates (years 1990–1992) to post-spill settlement rates (2010 and 2011), hypothesizing that post-spill rates would be lower at the oiled site (Dauphin) than at the un-oiled reference site (Galveston). Since the raw baseline data was lost in a hurricane (Rabalais, personal communication), we estimated 1990–1992 daily settlement rates from Fig six in Rabalais *et al*. [12] using the Engauage Digitizer (http://digitizer.sourcerorge.net/.). From our dataset, mean daily settlement rate was calculated by averaging daily settlement rates from the time periods covered in both the baseline and the current study (July 28^th^–October 31^st^). To test for significant spill effects, we conducted a factorial analysis of variance with Site (Galveston, Dauphin), Period (Before spill, After spill) and their interaction as fixed effects. We considered 2010 an After period because the well was temporarily capped on July 15^th^ 2010, prior to the first day of the time period analyzed (July 28^th^).

For the second DWH effects test, we compared the 2010 mean annual settlement rates and megalopal weights at sites exposed to DWH oil (Grand Isle, Rigolets, Ocean Springs, Dauphin, Pensacola) to those from sites not exposed to DWH oil (Galveston, Apalachicola). Oil exposure at each site was determined by maps of shoreline surveys from the Shoreline Cleanup Assessment Technique Program [5]. To test for DWH effects on mean megalopal settlement rates or weights, we calculated yearly daily settlement and weight averages for each site and conducted a one-way ANOVA with Oil status (Not Oiled, Oiled) as a fixed factor. For this analysis, settlement rates were log-transformed. Both analyses were conducted in the R version 3.1 [29].

Given low sample sizes due to limited baseline data and reference sites, we performed power analyses over a large range of effect sizes (0.1–0.8, or 10%-80% change in settlement rate) for both DWH effects tests. For the baseline comparison, we used a two-sample t-test power analysis, with 4 replicates in each group. For the between-site comparison test, we used the two-sample t-test power analysis with 2 replicates in the first sample (Not Oiled), and 5 replicates in the second sample (Oiled). All power analyses were conducted in the R pwr package [30].

### Time Series Modeling of Daily Settlement Rates

To assess the natural drivers of *C*. *sapidus* settlement in the GOM, we developed time series models to estimate the effects of seasonal, lunar, hydrodynamic and wind drivers on daily settlement within and between sites. This analysis included all four sampled sites from 2011 and five of the seven sampled sites from 2010, as two (Rigolets, Ocean Springs) did not have available buoy data. The physical drivers investigated were chosen because they have either been shown to or hypothesized to influence daily variation in *C*. *sapidus* megalopal settlement (Table 1).

The first step in this analysis estimated the role of seasonality in explaining daily settlement within and among sites. To do this we fit a seasonal sine-cosine function [sin(2π*Day*/365) +cos(2π*Day*/365), where *Day* represent the day since January of that year] to log-transformed daily settlement rates. To determine whether the seasonal trend varied by site or year, we fit models with and without Site, Year and their interaction terms and selected the best model using Akaike Information Criterion (AIC) [31].

The second step estimated lunar trends in settlement within and between sites. We did this by fitting seasonal model residuals to a sine-cosine function, [sin(2π*LunarDay*/30) +cos(2π*LunarDay*/30)] where *LunarDay* is the number of days since the last new moon. Again, AIC model selection was used to determine whether Site, Year or interaction terms were informative.

Finally, we estimated and removed any auto-correlation structure in the settlement data and then tested for hydrodynamic and wind effects on settlement. Using the residuals from the best lunar model, we first fit 1^st^ through 4^th^ order terms for autoregressive (AR), integrated (I) and moving average (MA) parts and used AIC plus visual inspection of residuals to determine the appropriate ARIMA structure. Residuals from the best ARIMA model were then used as the response variable in a linear regression with daily sea level flux, maximum sea level, mean night sea level, N-S wind component, year and all 2^nd^-order interactions of these variables as factors. Neither alongshore wind nor the E-W wind components were explanatory at any site, so we dropped these to reduce model complexity. We also explored lagged-regressions (up to 3 days lag), but these analyses did not yield any new information so are not presented here. All analyses were conducted in the R programming language [30], using the nls function for seasonal and lunar models, the arima function for the ARIMA structure estimation and the lm function for estimating hydrodynamic, wind and year factors. Percent variance explained by each factor (seasonal, lunar, ARIMA, hydrodynamic + wind variables) for each site was then calculated to determine the relative importance of the different drivers of megalopal settlement in this region.

## Results

### Daily Settlement and Bi-Weekly Weights in 2010 and 2011

As expected, *C*. *sapidus* daily settlement rate was highly variable within and among sites and years, with standard deviations between 1.5 and 6.5 times as large as yearly means (Table 2, S1 Fig). Settlement rates were highest by an order of magnitude in Pensacola (1786 individuals collector^-1^day^-1^ ± 3470 sd in 2010, 1594 ± 2202 in 2011), followed by Grand Isle (235 ± 1053 in 2010, 75 ± 130 in 2011), Dauphin (131 ± 656 in 2010, 73 ± 174 in 2011), Galveston (12 ± 43 in 2010, 3 ± 5 in 2011), Apalachicola (7 ± 13 in 2010), Ocean Springs (2 ± 5) and, finally, Rigolets (1.1 ± 7.1 in 2010). Mean daily settlement was always much higher than the median value, indicating that, as found in previous studies (Table 1), settlement was generally low most days but punctuated by a few days of high settlement (Table 2, S1 Fig). Within sites that were sampled in both 2010 and 2011, mean and maximum settlement was noticeably lower in 2011. However the number of pulse settlement events, which we defined as days with settlement greater than the mean plus two standard deviations, were generally similar except for at Grand Isle, where there were two pulses in 2010 and seven in 2011 (Table 2).

**Table 2:** Summary statistics of daily megalopal settlement across sites and years.

Site	Year	Start Date	Median	Mean	Max	Standard Deviation	Number of Pulses
Galveston	2010	05/27	2.0	12.3	310.0	43.0	4
Galveston	2011	05/17	1.5	3.0	28.5	4.5	5
Grand Isle*	2010	05/20	23.5	234.6	9665.0	1053.4	2
Grand Isle*	2011	05/16	18.8	75.4	902.5	130.0	7
Rigolets*	2010	05/05	0.0	1.1	69.0	7.1	2
Ocean Springs*	2010	05/10	0.3	2.2	38.5	4.7	4
Dauphin*	2010	05/19	21.0	130.6	6875.5	655.6	1
Dauphin*	2011	05/16	14.8	73.0	1221.3	173.8	3
Pensacola*	2010	05/18	536.9	1785.6	18301.0	3469.8	6
Pensacola*	2011	05/24	767.1	1594.0	14786.7	2202.4	4
Apalachicola	2010	06/28	2.8	6.7	76.5	12.9	3

The first day of sampling day at each site for each year is given as the Start Date, and end dates were all the last Friday in October of that year. Number of pulses was calculated as the number of days with settlement numbers above the mean + 2 SD. Median, mean and max units are all # *Callinectes spp*. megalopae collector^-1^ day^-1^. Daily settlement time series for each site and year are in S1 Fig.

*Indicates that site was considered within the spill extent.

Mean dry weights varied among sites and across years (S2 Fig), with highest mean dry weights found at Ocean Springs (0.32 mg individual^-1^ ± 0.06 sd in 2010), followed by Galveston (0.31 ± 0.05 in 2010, 0.25 ± 0.04 in 2011), Apalachicola (0. 28 ± 0.05 in 2010), Grand Isle (0.27 ± 0.05 in 2010, 0.29 ± 0.03 in 2011), Rigolets (0.26 ± 0.01 in 2010) and finally Pensacola (0.21 ± 0.03 in 2010, 0.20 ± 0.02 in 2011) and Dauphin (0.19 ± 0.02 in 2010, 0.21 ± 0.02 in 2011). Within a site, weights were generally lower in the warmer summer months, a pattern that corresponds to that of *Callinectes spp*. sizes observed by Ogburn *et al*. in both GOM and Atlantic coast samples [28].

### DWH Effects

**Fig 2:**
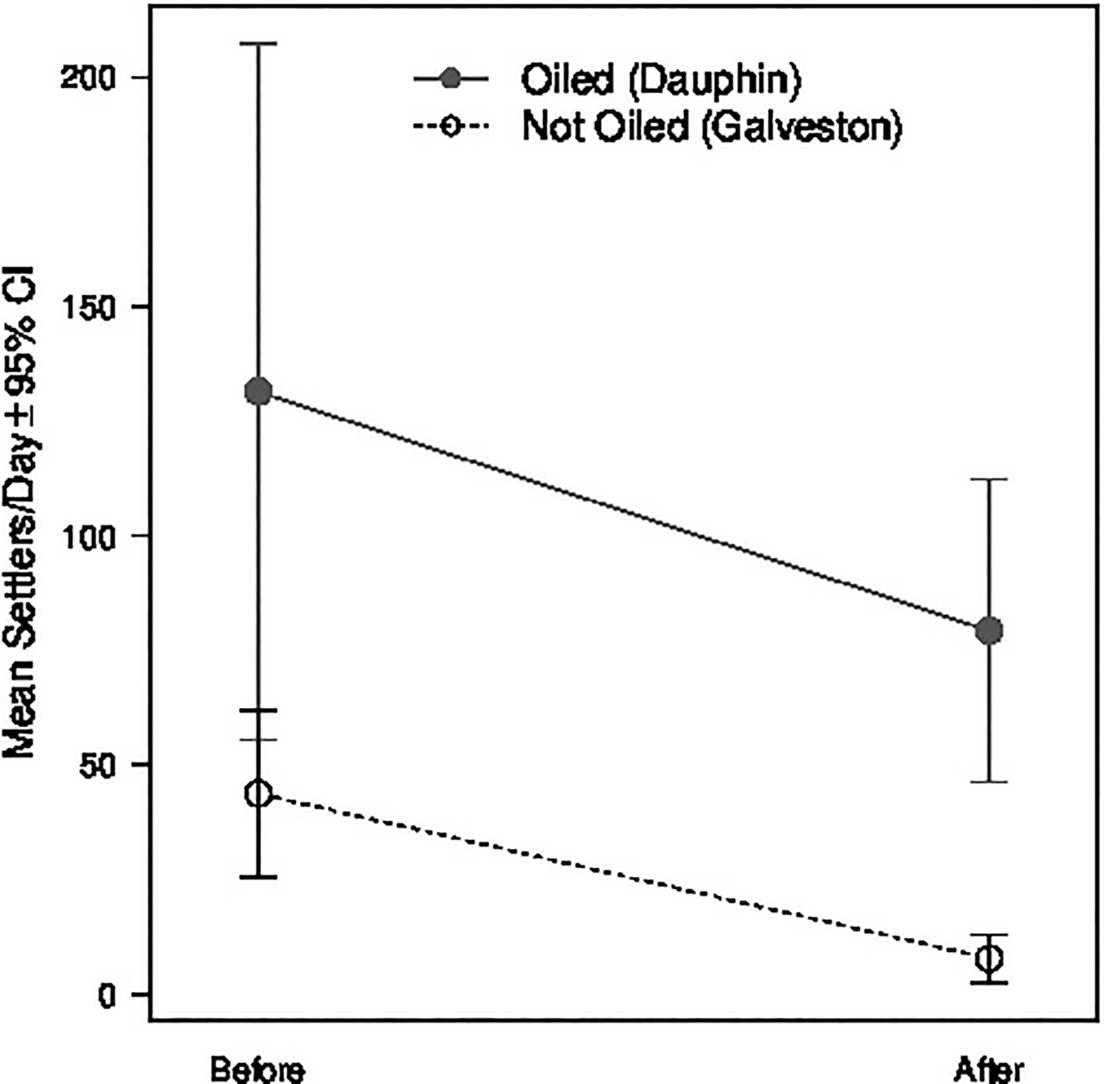
Megalopal settlement rates Before/After at Oiled/Not Oiled sites. Mean megalopal settlement rates Before (1990–1991 for Dauphin, 1991–1992 for Galveston) and After (2010–2011 for both Dauphin and Galveston) the Deepwater Horizon event at an Oiled site (Dauphin) and a Not Oiled site located well outside of the surface oil’s extent (Galveston). There were no significant differences in mean settlement between sites, time period (Before/After), or their interaction.

Comparison of mean settlement rates between an oiled and a not oiled site before and after the spill found no DWH effects on megalopal settlement (Fig 2). For Dauphin, the site within the spill zone, mean settlement rates for 1990 and 1991 were almost identical to those immediately after the DWH event in 2010 and 2011 (55.4 ± 103.8sd individuals collector^-1^day^-1^ in 1990, 207.4 ± 447.7 in 1991, 207.1 ± 864.5 in 2010 and 46.2 ± 76.3 in 2011). For Galveston, the site outside of the spill zone, mean daily settlement rates were 25.5 ± 58.2 individuals collector^-1^day^-1^ and 62.0 ± 168.0 in 1991 and 1992, respectively. While mean settlement rates in 2010 and 2011 were lower (13.0 ± 47.6 and 2.6 ± 3.1 respectively), they were still well within the range of natural variation. ANOVA results found no significant effects of site, year or a site by year interaction (Table 3).

**Table 3:** Analysis of variance for the effect of Oiled/Not Oiled and Before/After DWH on megalopal settlement.

Variable	df	Sum of Squares	Mean Square	F-value	p-value
Site	1	12,664.4	12,664.4	3.50	0.13
Period	1	3,876.4	3,876.4	1.07	0.36
Site*Period	1	130.4	130.4	0.03	0.86
Residuals	4	14,463.4	3,615.4		

The variables tested were Site (Dauphin = Oiled, Galveston = Not Oiled), Period (Before/After DWH) and their interaction (Site*Period). df = degrees of freedom. No significant effects were found (p>0.05 for all variables).

**Fig 3:**
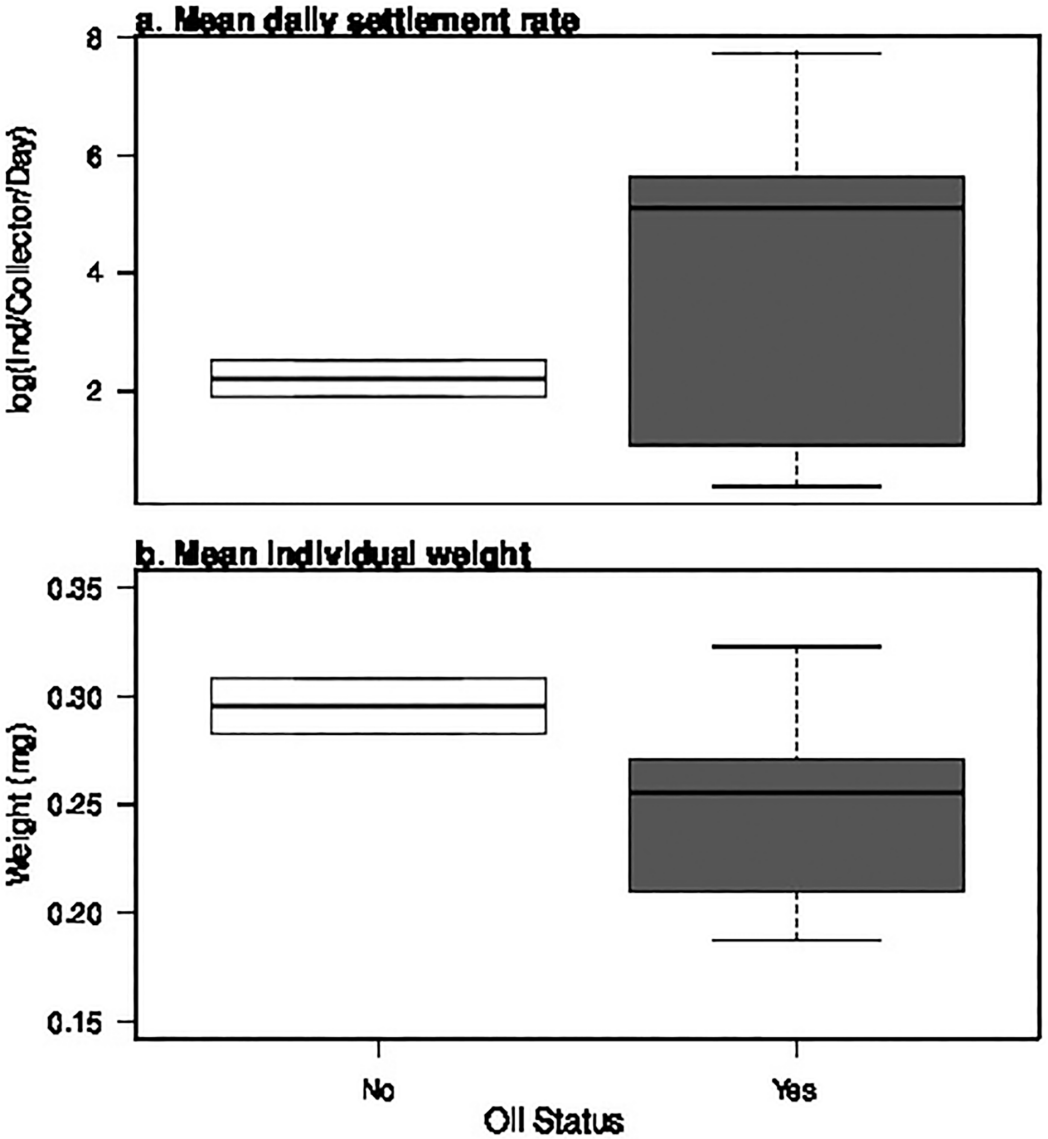
Megalopal settlement rates and weights at Oil/Not Oil sites in 2010. (a) Mean daily settlement rate and (b) mean individual weight of *Callinectes spp*. megalopae at Not Oiled (white, box, n = 2) and Oiled sites (grey box, n = 5) in 2010. Boxplots show the median (black line), the first and third quartiles (lower and upper box bounds, respectively) and 95% confidence intervals (whiskers). For the Not Oiled sites (Galveston, Apalachicola), the whiskers are right on the bounds of the box. There was no significant effect of oil status on either variable.

Comparison of megalopal settlement rates and dry weights between Oiled and Not Oiled sites in 2010 also failed to find evidence of a DWH effect on settlement or dry weight (Fig 3 and S3 Fig, ANOVA Oil effect on settlement rate: df = 1, SS = 4.539, F-val = 0.573, p = 0.483; ANOVA Oil effect on megalopal weight: df = 1, SS = 0.003, F = 0.307, p = 0.305).

The statistical power for each test was found to be low, even for large effect sizes. For the baseline comparison test, the power to detect over the range of effect sizes (0.1–0.8, or 10%-80% difference) ranged from 0.052–0.16, indicating that we only had a 16% likelihood of detecting an 80% change in megalopal settlement rates. For the between-site comparison test, the power ranged from 0.051–0.123 over the same range of effect sizes, again indicating low power to detect even large effect sizes (e.g., only a 12.3% likelihood of observing an 80% change in settlement rates).

### Time Series Modeling

**Fig 4:**
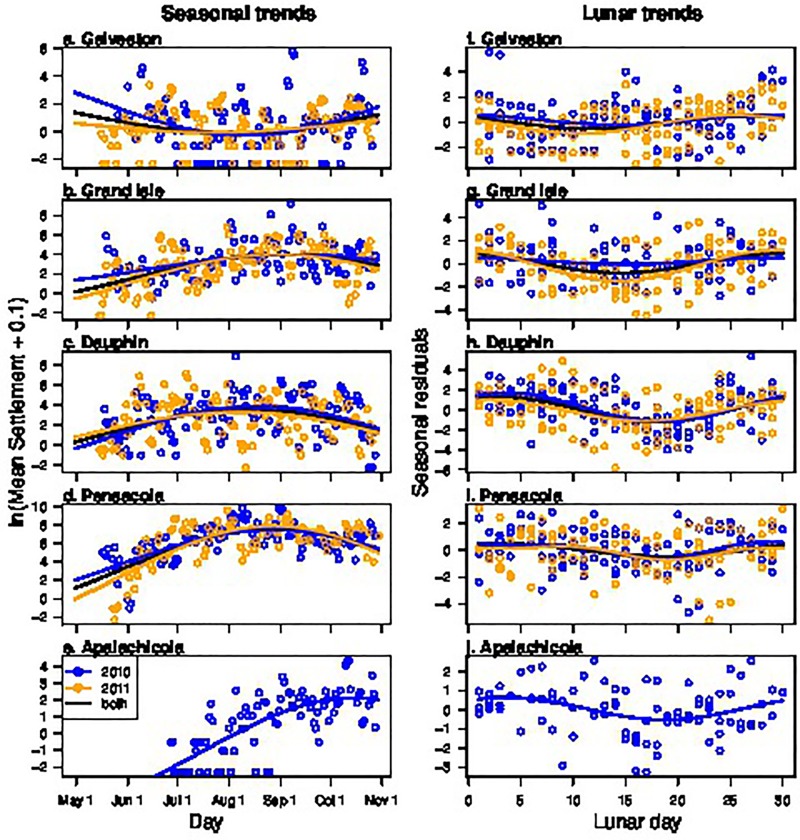
Seasonal (a-e) and lunar (g-j) trends for each site in 2010 (blue) and 2011 (orange). The black line represents the average trend over the two years. For both seasonal and lunar trend analysis, the Site-only and full-factorial (Site*Year) models were equivalent, and we have displayed the Site-only model for simplicity. Apalachicola was only sampled in 2010.

The seasonal trend in settlement was different among sites but very similar within a site across years 2010 and 2011 (Fig 4A–4E, Table 4A). The full factorial model and the model with only site as a factor (“Site-only” model) were found to be equivalent by AIC, meaning that the variance explained by Year was negligible. Most sites had a peak in settlement from mid-August to early September (Grand Isle–Sep 3^rd^, Dauphin Aug 13^th^, Pensacola Aug 28^th^), while peak settlement at Apalachicola occurred much later (Oct 16^th^) and settlement at Galveston exhibited a gradual decline from a peak on Apr 30^th^ to a minimum on Aug 3^rd^. Downstream analyses using residuals from the full-factorial and Site-only seasonal models were similar, so we only present the Site-only model for simplicity.

**Table 4:** AIC scores for seasonal and lunar trend models.

**a. Seasonal trend models**	**k**	**AIC**	**ΔAIC**
sin(2πDay/365)*Site*Year + cos(2πDay/365)*Site*Year	28	3769.3	0
sin(2πDay/365)*Site + cos(2πDay/365)*Site	16	3769.4	0.1
sin(2πDay/365)*Year + cos(2πDay/365)*Year	7	4704.8	935.6
sin(2πDay/365) + cos(2πDay/365)	4	4707.3	938.0
null	2	4741.1	971.9
**b. Lunar trend models**			
sin(2πLunarDay/30)*cos(2πLunarDay/30)*Site*Year	37	3645.2	0
sin(2πLunarDay/30)*cos(2πLunarDay/30)*Site	21	3645.5	0.4
sin(2πLunarDay/30)*cos(2πLunarDay/30)*Year	9	3656.9	11.7
sin(2πLunarDay/30)*cos(2πLunarDay/30)	5	3663.4	18.3
null	2	3741.4	96.2

The AICs are ranked lowest to highest for the (a) seasonal and (b) lunar trend models. The variables, number of parameters (k), Akaike information criterion (AIC), and the difference from the lowest AIC score are presented for each model (ΔAIC). Models with AICs within 2 points are considered equivalent.

Lunar trends were also found to be different among sites but similar within sites across years (Fig 4F–4J, Table 4B) and the full-factorial and Site-only lunar models were found to be equivalent. Lunar trends appeared most similar among sites on either side of the Mississippi River. The three sites east of the Mississippi River Delta had higher settlement in the days immediately following the new moon (Dauphin maximum settlement rate at Lunar Day = 3, Pensacola at 3, Apalachicola at 4), which declined until a few days following the full moon (Dauphin minimum at Lunar Day 18, Pensacola at 19, and Apalachicola at 18), after which settlement began increasing again. The sites west of the Mississippi River Delta had peak settlement just before the new moon (Galveston at Lunar Day 27, Grand Isle at 30), which declined to a minimum just prior to the full moon (Galveston at Lunar Day 11, Grand Isle at 14). Again, downstream analyses for both the full-factorial and the Site-only lunar models were similar, so we present the Site-only model results for simplicity.

At all sites, the best ARIMA structure of the lunar residuals included a 1^st^-order autoregressive term and no integrated or moving average terms, except for Grand Isle which required a 2^nd^ order moving average term. After factoring out these ARIMA structure for each site, hydrodynamic and wind factors were found to be variable across sites (Table 5). There were no significant variables at Galveston. At Grand Isle, settlement decreased with daily sea level flux, contrary to expectations, but increased with an increasing flux by night sea level interaction as expected. At Dauphin, the only significantly explanatory variables were interactions between daily max sea level and the N-S wind component, between night sea level and the N-S wind component, and a slightly stronger night sea level effect in 2011 than in 2010. At Pensacola, settlement was influenced only by a N-S wind component effect that was stronger in year 2011 than in 2010. At Apalachicola, settlement increased with daily sea level flux as expected.

**Table 5:** Results of the hydrodynamics and wind multiple linear regressions.

	Flux	Max	NS	Night	Year	Flux * Max	Flux * N-S	Flux * Night	Flux * Year	Max * NS	Max * Year	NS * Night	NS * Year	Night * Year
Galveston	-2.9	-3.1	-0.1	-3.0	-1.2	7.2	0.2	7.9	2.4	—	—	—	—	—
Grand	**-2.3**	—	-0.2	-5.9	—	—	0.4	**13.2**	—	—	—	—	—	—
Dauphin	-0.6	-0.7	-0.1	-3.4	-0.7	—	-0.7	—	—	**0.8**	—	**0.6**	—	**3.7**
Pensacola	0.8	-0.6	-0.1	—	-0.2	—	—	—	-2.5	—	3.7	—	**0.2**	—
Apalachicola	**2.2**	—	—	—	NA	—	—	—	NA	—	NA	—	NA	NA

For each site, coefficients of the best model (as determined by AIC) that were significant at the p<0.05 level are given in bold. For site Apalachicola, terms involving Year are marked “NA” as this site only had data from 2010 and “—”indicates that a variable was not present in the best model. Flux = Daily Sea Level Flux (m), Max = Daily Maximum Sea Level Height (m), N-S = North-South wind component (m s^-1^), Night = Mean Sea Level at Night (m) and “*” indicates an interaction between terms. Max*Night interactions were not in any best model, so this term was omitted from the table.

**Fig 5:**
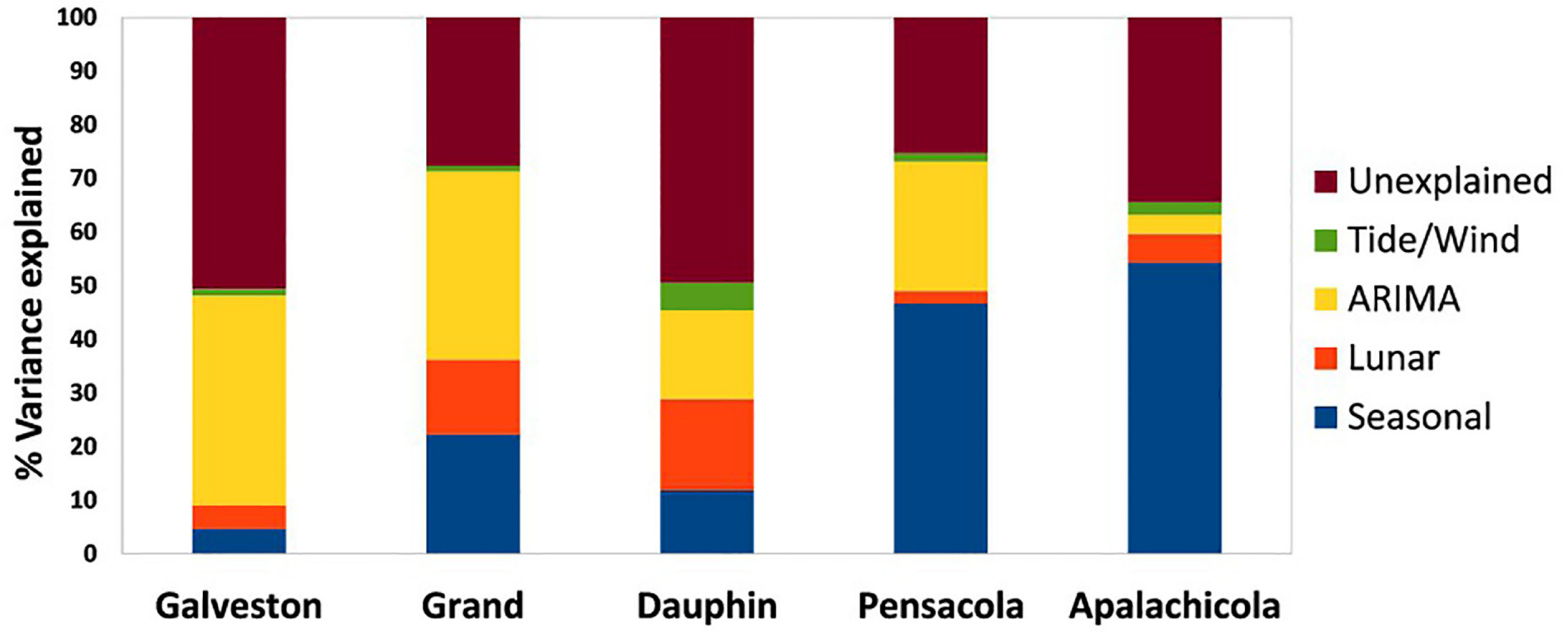
Variance in settlement explained by each physical driver by site. Variance explained was determined by fitting the best model for each factor for each site, then dividing the residual variance by the variance in the raw data.

Overall the seasonal, lunar and ARIMA factors consistently explained more variation in settlement than hydrodynamic or wind factors (Fig 5). On average, seasonal trends explain the most variance (mean = 28.0%), followed by autoregressive structure (mean = 23.7%), lunar trend (mean = 8.6%) and hydrological/meteorological effects (mean = 2.3%). After accounting for all of these factors, an average of 37.5% variation remained unexplained across sites. Galveston had the most unexplained variance (50.6%), followed by Dauphin (49.4%), Apalachicola (34.5%), Grand Isle (27.4%), and Pensacola (25.3%).

This analysis fit seasonal and lunar trends before testing for hydrodynamic and wind effects as is standard for most time series analyses [32]. However, since our hydrodynamic and wind variables were likely to exhibit seasonal or lunar trends similar to those found in settlement rates, it is possible that this approach could have masked important hydrodynamic or wind effects. To explore this possibility, we fit seasonal and lunar trends to all of the hydrodynamic and wind variables using the methods described above. None of the variables exhibited a prominent seasonal trend but many hydrodynamic variables displayed lunar trends similar to those found in megalopal settlement (S1 File). To explore how this cross-correlation might change our results, we fit linear models estimating the effects of hydrodynamic and wind factors and all of their second-order interactions to the raw settlement data, the residuals from the seasonal model, and the residuals from the season and lunar models (S1 Table). These results found that the overall explanatory power of the hydrodynamic and wind variables depended on which step in the analysis they were considered. On average, across sites, hydrodynamic and wind factors explained 7.8% of the variance when autoregressive structure was not removed (but seasonal and lunar trends were removed), 9.9% of the variance when neither autoregressive nor lunar trends were removed (but seasonal trends were removed), and 17.9% of the variance when neither autoregressive, lunar, nor seasonal trends were removed (raw data). However, similar to our initial analysis, no single hydrodynamic or wind variable had a consistent effect across sites nor did they explain more variance than the seasonal, lunar and ARIMA factors. Therefore, since our overall interpretations of these effects remain the same, we chose to present only the initial time series analysis (e.g., first removing the seasonal trend, then the lunar trend, then ARIMA structure, and finally hydrodynamic and wind factors). Time series plots of *Callinectes spp*. settlement rates with the hydrodynamic and wind variables are available as S2 File.

## Discussion

This study found no evidence of a decline in Blue Crab megalopal settlement or condition associated with the Deepwater Horizon oil spill. No significant difference in mean annual settlement was observed in 2010 at two sites with baseline data from the early 1990s and settlement rates and weights were similar in 2010 and 2011 in sites inside and outside of the DWH surface oil’s extent. However, our power analysis showed that detecting even large effects would have been unlikely given the large amount of unexplained variation in yearly and daily settlement rates and the paucity of baseline data. For example, the probability of detecting an 80% change in mean megalopal settlement would have been only 16% in the Before/After site comparison and only 13% in the 2010 Not Oiled/Oiled site comparison. Additionally, mean and maximum settlement values tended to be lower in 2011 compared to 2010, potentially due to lagged or indirect effects of the DWH.

In addition to low statistical power which could be masking a true DWH effect, we further caution that one or more of our assumptions could be wrong. The first assumption, that Blue Crab larvae came in contact with DWH oil at the “Oiled” sites (Grand Isle, Rigolets, Ocean Springs, Dauphin, Pensacola) and not at the “Not Oiled” sites (Galveston, Apalachicola), could be incorrect in two ways. First, the Blue Crab larvae from the Oiled sites may have been able to detect and behaviorally avoid oil as adult Blue crabs and many zooplankton are known to do [33–35]. Alternatively, our simple delineation of Oiled and Not Oiled sites based on surface oil maps might not reflect the dispersal of oiled Blue Crab zoea throughout the GOM. Previous studies suggest that oiled Blue Crab megalopae would settle near to their spawning sites, including a detailed consideration of Blue Crab larval biology and seasonal circulation patterns in the Mississippi Bight [36] as well as a recent Blue Crab particle-tracking study covering the entire northern GOM [37]. However, chemical signatures from the DWH spill in the waters, sediments and organisms have been found as far away as Galveston [38], indicating that widespread dispersal of DWH oil and oiled Blue Crab larvae could have occurred, which would make our local retention assumption erroneous.

Our second assumption, that oil or oil-dispersant mixture had negative effects on Blue Crab larvae, could be false if Blue Crab larvae are highly tolerant or if oil concentrations in the surface waters were not high enough to cause immediate harm. Even though several studies have shown that oil-dispersant mixtures are toxic to Blue Crab juveniles, megalopae and zoeae [6–9], juvenile crabs are relatively highly tolerant of oil or dispersant compare to other estuarine organisms [39, 40] and near-surface zooplankton communities have been observed to recover quickly post DWH spill [41]. This suggests that toxic levels oil-dispersant mixtures either never manifested during the spill [9] or were quickly diluted, either by wave action or by rapid microbial degradation, thereby only affecting larvae that were immediately present at the time of dispersant application.

Our third assumption, that mortality and growth rates would have been similar in the 2010 compared to the baseline years, is almost certainly false given the complexity of the oil’s effect on a food web and of the human response to the spill. For example, any negative effects of oil may have been offset by positive effects of oil carbon to the planktonic food-web [42] or because predatory fish avoided oiled areas, thereby increasing larval survivorship and growth rates. Additionally, the human responses to the spill may have had complex effects on the GOM ecosystem and hydrodynamics, all of which could have influenced background mortality or settlement rates in 2010. For example, a federal fishing ban covered up to 36.6% of the GOM Exclusive Economic Zone at its peak and lasted until November 15^th^, 2010 in some areas [43], likely reducing fishing pressure on Blue Crabs but also increasing predation pressure by some the crab’s natural predators. In Louisiana, diversions of the Mississippi River meant to prevent the influx of oil into bays and marshes temporarily reduced salinities [44], negatively affecting oysters [45] but with unknown impacts on Blue Crabs.

Finally, our knowledge of Lesser Blue Crab (*C*. *similis*) settlement patterns, Blue Crab spawning biology and inter-annual variation in spawning stock size is incomplete, potentially misleading our interpretations. Megalopae of *C*. *similis* have been collected at coastal GOM sites similar to those sampled in this study and they are larger than but easily confused with those of *C*. *sapidus* [28, 46]. However, *C*. *similis* megalopal abundances in coastal GOM waters have been observed to peak in March and be much lower than that of *C*. *sapidus* throughout the rest of the year [47], indicating that potential *C*. *similis* megalopae in our May-December samples would likely alter total settlement numbers only slightly. Consistent with our spawning assumptions, recent spawning grounds have been documented on shoals offshore of Louisiana [4], and hydrodynamic simulations have found that Blue Crab megalopae are likely to settle near their spawning site in the northern GOM [36, 37], indicating that Blue Crab eggs and zoea would have likely settled near the point of oil contact. With respect to hydrodynamics, Loop Current intrusions and associate eddies are thought to influence Blue Crab dispersion and settlement success [36], so variation in these large-scale features could have caused variation in Blue Crab dispersal patterns between years. Eddy spin-offs have been observed to become more frequent from 2001–2010 [48], potentially confounding our baseline (1990–1992) and 2010 comparisons. Finally, the assumption that spawning stock sizes were similar across years is difficult to assess. Commercial landings have been slowly declining in the GOM from the early 1990s to 2013 (S4 Fig), however these data cannot be easily translated into stock sizes because they do not account for changes in fishing effort or external factors such as the 2010 federal fishery closure. Fishery-independent estimates of juvenile and adult Blue Crab abundance also reveal a steady decline in the western GOM (Apalachicola to Texas) from the mid-1980s to the mid-1990s, after which abundances leveled off [49]. This is consistent with our observation that settlement tended to be lower at both Dauphin (Oiled site) and Galveston (Not Oiled site) in 2010–2011 compared to 1990–1992 (Fig 2). Given these uncertainties, further research into basic biology and ecology of GOM Blue Crabs, specifically their spawning biology, inter-annual larval dispersion and stock size dynamics, and toxicity studies under more realistic conditions over all life stages, is needed to better assess these alternate hypotheses regarding DWH effects on GOM Blue Crabs.

Like this study, several others have also found that Blue Crabs were not severely impacted by the DWH in the short term. Blue Crab settlement in Mississippi during the DWH was found to be similar to that in baseline years [9] and juvenile Blue Crab abundance in Alabama marshes was significantly lower in 2010 but had rebounded by 2011 [50]. In light of the megalopae findings in this study and [9], it appears that the 2010 decline in Alabama marshes was more likely caused by post-settlement processes such as increased predation or indirect oil effects, and not megalopal supply. The difficulty in detecting population-level impacts of an oil spill can be due to the lack of baseline data, the complex spatio-temporal dynamics of any ecosystem, and the fact that, as previous oil spill research has shown, sub-lethal and indirect impacts of an oil spill can take years or decades to manifest [42]. The trend towards lower mean and maximum daily settlement rates in 2011 at four GOM site (Table 2, S1 Fig) warrants continued monitoring of the GOM Blue Crab population for potential long-term impacts of the spill.

In addition to DWH effects, this study also contributes to our understanding of natural drivers of Blue Crab settlement in the GOM. Our time series analysis found that seasonal and lunar trends were very similar across years within a site and also generally similar across sites east of the Mississippi River Delta with peaks in August-early September and shortly after the new moon. Together, seasonal and lunar trends explained a large amount of variation (29–60%) at each site except Galveston, where only 9.1% of the variation was explained by these variables. Galveston, the extreme western site, exhibited a different seasonal pattern from the eastern sites, suggesting that megalopal settlement dynamics may be driven by different factors in the western GOM. This finding is consistent with a particle-tracking study which found that the Mississippi River acts as a barrier to Blue Crab zoeae dispersal [37]. Previous studies from the GOM (Table 1) have also found settlement peaks in August-early September and higher settlement in either the first [12] or third [20] lunar phases, corresponding to the new and full moons, although several studies found no differences in lunar phases [18, 19, 21]. Since most of these studies tested for lunar effect by categorizing into 4 lunar phases, it could be that artifacts of this categorization or failure to seasonally de-trend the data masked a real lunar trend. It could also be that lunar trends are truly negligible at some sites or during some years. However, given the consistency of the lunar trends east of the Mississippi River Delta and within sites over years, we suggest that lunar synchronicity in spawning, egg hatching or larval development is an important driver of Blue Crab settlement dynamics in this region. In North Carolina, female ovigerous crabs exhibit circatidal swimming and abdominal pumping rhythms that are thought to promote spawning synchronicity during morning ebb tides. [51, 52]. Whether these rhythms are similar in the Gulf of Mexico, which has diurnal tides opposed to the semidiurnal tides of the western Atlantic, remains unknown.

Contrary to our expectations, we found that hydrodynamic and wind factors were inconsistent and relatively weak across the GOM (Table 5, Fig 5). This could explain the fact that, despite the focus on hydrodynamic and wind factors in previous studies (Table 1), little agreement has been reached concerning their importance in settlement. For example, both Morgan et al. [19] and Spitzer et al. [21] found that wind velocity explained daily settlement rates in Mobile Bay, but such effects were not found in other GOM sites [12, 18, 20]. Some of these incongruences can be explained by considering the geography of the different sites. For example, Hasek and Rabalais [20] found that megalopal settlement increased with maximum sea level in a Louisiana marsh, but this site was far inland where settlement is likely driven by megalopal tidal-stream transport processes [3] rather than the coastal settlement processes that were the focus of this study. However, most other GOM sites in this study could be considered coastal, and even those did not find consistent hydrodynamic or wind effects. Given the different statistical methods used by each study, and the fact that no previous GOM study has accounted for seasonal and autoregressive effects, it is unclear if these effects truly vary between sites and times or if statistical analyses produced artifacts. Additional time-series data spanning more sites and analyzed in a consistent manner, and including partitioning of variance for each factor, would help clarify the important drivers of Blue Crab settlement in the GOM and enable us to better detect perturbations.

Overall, this study makes an important first step in evaluating DWH effects on and understanding natural drivers of GOM Blue Crab settlement. Given the low power of the analyses possible with available data, we can only conclude that the DWH did not result in extreme Blue Crab megalopal mortality, as both settlement rates and megalopal weights during the DWH appear within the limits of naturally high variation. A trend towards lower mean settlement and lower maximum settlement in 2011 at four sites warrants continued monitoring of GOM Blue Crabs for any long-term DWH effects. In exploring natural drivers of settlement, we found that seasonal and lunar trends are remarkably consistent within sites and that hydrodynamic and wind effects were inconsistent. This suggests that synchronicity in spawning, hatching or larval development at seasonal and lunar frequencies could be driving megalopal settlement patterns. This finding should motivate more research to understand Blue Crab spawning biology in the GOM, which is currently poorly known. Further, the general discordance in trends between sites east and west of the Mississippi River Delta suggests that dispersal ecology may be fundamentally different in these two regions, a question that could be addressed with further empirical research and continued monitoring.

## Supporting Information

S1 Fig Mean daily settlement rates by site over time. (DOCX)

S2 Fig Megalopal dry weights by site over time. (DOCX)

S3 Fig Box plots of 2010 megalopal settlement rates and dry weights by site. (DOCX)

S4 Fig Commercial landings of Blue Crabs in Gulf of Mexico from 1989–2013. (DOCX)

S1 Table Alternate time series analyses. (DOCX)

S1 File Seasonal and lunar trends in hydrodynamics and wind variables. (DOCX)

S2 File Time series plots of *Callinectes spp*. settlement rates, hydrodynamic and wind variables. (DOCX)
