# Supplementary material for: Evaluation of Blue Crab, Callinectes sapidus, Megalopal Settlement and Condition during the Deepwater Horizon Oil Spill
Source: PLoS One. 2015 Aug 13;10(8):e0135791. doi: 10.1371/journal.pone.0135791 (PMC4535880; doi:10.1371/journal.pone.0135791)
Supplement: S1 Fig — (DOCX) [file pone.0135791.s001.docx]

**Supplement 1 – Mean daily settlement rates by site over time.**

Mean daily settlement rate over time at each site. Mean rates were calculated by averaging over three to four collectors per site. 2010 data are shown in blue and 2011 data are shown in orange. Error bars represent standard errors.
