# Supplementary material for: Evaluation of Blue Crab, Callinectes sapidus, Megalopal Settlement and Condition during the Deepwater Horizon Oil Spill
Source: PLoS One. 2015 Aug 13;10(8):e0135791. doi: 10.1371/journal.pone.0135791 (PMC4535880; doi:10.1371/journal.pone.0135791)

**Supplement 2 – Megalopal dry weight by site over time.**

Megalopal dry weight (mg) over time at seven sites in 2010 (solid blue bar) and four sites (Galveston, Grand Isle, Dauphin, and Pensacola) in 2011 (striped orange bar). Weights were estimated by pooling 20 or 100 individuals from each site over two week periods from May 15^th^ to October 31^st^.


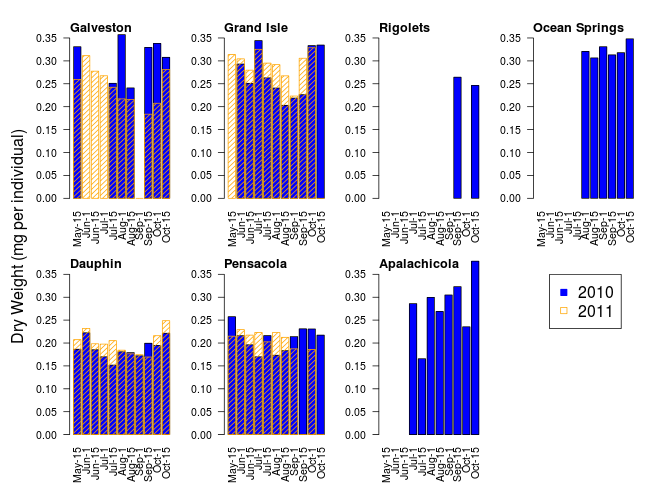

Supplement: S2 Fig — (DOCX) [file pone.0135791.s002.docx]
