# Supplementary material for: Evaluation of Blue Crab, Callinectes sapidus, Megalopal Settlement and Condition during the Deepwater Horizon Oil Spill
Source: PLoS One. 2015 Aug 13;10(8):e0135791. doi: 10.1371/journal.pone.0135791 (PMC4535880; doi:10.1371/journal.pone.0135791)

**Supplement 3 – Box plots of 2010 megalopal settlement and dry weight by site.**

Mean megalopal settlement rates (# individuals per collector per day) and individual dry weights (mg) at all seven sites sampled in 2010. White boxes represent No Oil sites and grey boxes represent Oil sites. The black bar represents the median, the box represents 1^st^ and 3^rd^ quartiles, the whiskers represent +/-95% confidence intervals and the circles represent outliers.


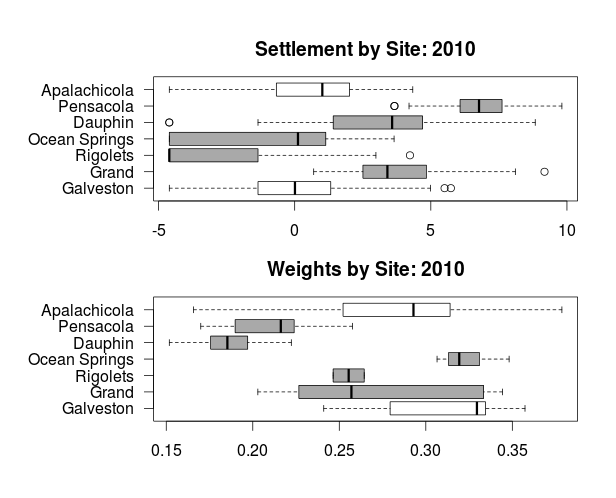

Supplement: S3 Fig — (DOCX) [file pone.0135791.s003.docx]
