# Supplementary material for: Evaluation of Blue Crab, Callinectes sapidus, Megalopal Settlement and Condition during the Deepwater Horizon Oil Spill
Source: PLoS One. 2015 Aug 13;10(8):e0135791. doi: 10.1371/journal.pone.0135791 (PMC4535880; doi:10.1371/journal.pone.0135791)
Supplement: S4 Fig — (DOCX) [file pone.0135791.s004.docx]

**Supplement 7 – Commercial Blue Crabs Landings in the GOM: 1989-2013**

Commercial landings of Blue Crab (in metric tons) from 1989-2013. Data were downloaded from NOAA (National Oceanic and Atmospheric Administrations) Annual Landings Statistics (<http://www.st.nmfs.noaa.gov/commercial-fisheries/commercial-landings/annual-landings/index>) downloaded on July 8^th^, 2015. Soft shell and peeler data were not included in this dataset. Overall Gulf of Mexico landings (a), State-wide landings in Texas (b), Louisiana (c), Alabama (d), Mississippi (e) and the Florida West (GOM) Coast (f).
