# Supplementary material for: Evaluation of Blue Crab, Callinectes sapidus, Megalopal Settlement and Condition during the Deepwater Horizon Oil Spill
Source: PLoS One. 2015 Aug 13;10(8):e0135791. doi: 10.1371/journal.pone.0135791 (PMC4535880; doi:10.1371/journal.pone.0135791)
Supplement: S1 Table — (DOCX) [file pone.0135791.s005.docx]

**Supplement 5 Table - Alternate Time Series Analyses**

To investigate how hydrodynamic and wind effects might change depending on the order of the time series analysis, we performed regressions on season + lunar + ARIMA residuals (a), season + lunar residuals (b), just season residuals (c), and the raw settlement data (d). For each analysis, we report the best fit model per site, the coefficient values of the best model, and the percent variance explained by the best model. Significant effects, as determined by ANOVA, are in bold with significance level (*p<0.5, **p<0.01, ***p<0.001). For Apalachicola, all Year terms are “NA” as this site was only sampled in 2010. A “--“ indicates the effect is not present in the best model. No model had a Max by Night interaction term, so it was dropped from the tables.

| 1. **SEASON + LUNAR + ARIMA RESIDUALS (Presented in Manuscript)** | | | | | | | | | | | | | | | | | | | | | | | | | | | | |  | | |  |
| --- | --- | --- | --- | --- | --- | --- | --- | --- | --- | --- | --- | --- | --- | --- | --- | --- | --- | --- | --- | --- | --- | --- | --- | --- | --- | --- | --- | --- | --- | --- | --- | --- |
|  | Flux | | Max | | | | NS | | Night | | Year | | Flux X Max | | Flux X N-S | Flux X Night | | Flux X Year | | Max X NS | | Max X Year | | NS X Night | | NS X Year | | Night X Year | | | **%Variance Explained** | |
| Galveston | -2.9 | | -3.1 | | | | -0.1 | | -3.0 | | -1.2 | | 7.2 | | 0.2 | 7.9 | | 2.4 | | -- | | -- | | -- | | -- | | -- | | | 1.2% | |
| Grand | **-2.3*** | | -- | | | | -0.2 | | -5.9 | | -- | | -- | | 0.4 | **13.2*** | | -- | | -- | | -- | | -- | | -- | | -- | | | 1.0% | |
| Dauphin | -0.6 | | -0.7 | | | | -0.1 | | -3.4 | | -0.7 | | -- | | -0.7 | -- | | -- | | **0.8*** | | -- | | **0.6*** | | -- | | **3.7*** | | | 5.3% | |
| Pensacola | 0.8 | | -0.6 | | | | -0.1 | | -- | | -0.2 | | -- | | -- | -- | | -2.5 | | -- | | 3.7 | | -- | | **0.2*** | | -- | | | 1.5% | |
| Apalachicola | **2.2*** | | -- | | | | -- | | -- | | NA | | -- | | -- | -- | | NA | | -- | | NA | | -- | | NA | | NA | | | 2.4% | |
|  |  | |  | | | |  | |  | |  | |  | |  |  | |  | |  | |  | | Average Variance | | | | | | | 2.28% | |
| 1. **SEASON + LUNAR RESIDUALS** | | | | | | | | | | | | | | | | | | | | | | | | | | | | |  | | |  |
|  | Flux | Max | | | | | NS | | Night | | Year | | Flux X Max | | Flux X N-S | Flux X Night | | Flux X Year | | Max X NS | | Max X Year | | NS X Night | | NS X Year | | Night X Year | | | **%Variance Explained** | |
| Galveston | **-2.5*** | | | -- | | | -0.1 | | **-5.7*** | | -1.7 | | -- | | -- | **14.5*** | | 4.8 | | -- | | -- | | -- | | **0.2**** | | -- | | | 11.3% | |
| Grand | **-2.7**** | | | -2.4 | | | -0.5 | | -7.2 | | -0.6 | | -- | | **1.1*** | 14.0 | | -- | | -- | | -- | | -- | | -- | | -- | | | 7.3% | |
| Dauphin | 0.6 | | | -2.0 | | | -0.1 | | **-4.7*** | | -1.1 | | -- | | -0.9 | -- | | -3.7 | | **0.9**** | | 5.1 | | **0.9**** | | -- | | **4.9**** | | | 11.2% | |
| Pensacola | **2.8**** | | | -1.5 | | | **-0.04*** | | -- | | -0.2 | | -- | | -- | -- | | -4.0 | | -- | | **5.8*** | | -- | | **0.3**** | | -- | | | 5.6% | |
| Apalachicola | **2.8*** | | | -- | | | -- | | -- | | NA | | -- | | -- | -- | | NA | | -- | | NA | | -- | | NA | | NA | | | 3.4% | |
|  |  |  | | | | |  | |  | |  | |  | |  |  | |  | |  | |  | | Average Variance | | | | | | | 7.76% | |
| 1. **SEASON RESIDUALS** | | | | | | | | | | | | | | | | | | | | | | | | | | | | |  | | |  |
|  | Flux | Max | | | | | NS | | Night | | Year | | Flux X Max | | Flux X N-S | Flux X Night | | Flux X Year | | Max X NS | | Max X Year | | NS X Night | | NS X Year | | Night X Year | | | **%Variance Explained** | |
| Galveston | -6.4 | | | | -3.6 | | -0.1 | | **-5.7*** | | -1.5 | | 10.7 | | -- | **14.5*** | | 5.1 | | -- | | -- | | -- | | **0.2**** | | -- | | | 12.9% | |
| Grand | **-2.9***** | | | | -3.0 | | -0.5 | | -6.5 | | **-0.9**** | | -- | | **1.2*** | 12.9 | | -- | | -- | | -- | | -- | | -- | | -- | | | 6.5% | |
| Dauphin | 6.6 | | | | 0.6 | | -0.1 | | **-6.7*** | | -1.3 | | -12.4 | | -0.8 | -- | | -7.2 | | **0.7*** | | **9.5**** | | **0.9**** | | -- | | **7.8***** | | | 15.4% | |
| Pensacola | **3.4**** | | | | -2.0 | | -0.1 | | -- | | -0.3 | | -- | | -- | -- | | -4.5 | | -- | | **6.9*** | | -- | | **0.4***** | | -- | | | 7.3% | |
| Apalachicola | **3.3***** | | | | 1.8 | | -- | | -- | | NA | | -- | | -- | -- | | NA | | -- | | NA | | -- | | NA | | NA | | | 7.8% | |
|  |  |  | | | | |  | |  | |  | |  | |  |  | |  | |  | |  | | Average Variance | | | | | | | 9.99% | |
| 1. **RAW DATA** | | | | | | | | | | | | | | | | | | | | | | | | | | | | |  | | |  |
|  | Flux | Max | | | | NS | | Night | | Year | | Flux X Max | | Flux X N-S | | Flux X Night | Flux X Year | | Max X NS | | Max X Year | | NS X Night | | NS X Year | | Night X Year | | | **%Variance Explained** | |  |
| Galveston | **-7.3*** | -4.0 | | | | -0.1 | | -5.2 | | -1.8 | | 11.8 | | -- | | 16.1 | 6.2 | | -- | | -- | | -- | | **0.2**** | | -2.5 | | | 16.6% | |  |
| Grand | -4.24 | -- | | | | **-0.3*** | | -2.9 | | **-1.2***** | | -- | | 0.8 | | -- | -- | | -- | | -- | | -- | | -- | | -- | | | 17.7% | |  |
| Dauphin | 0.11 | **-0.6*** | | | | 0.03 | | **-7.3**** | | -1.2 | | -- | | -1.0 | | -- | -5.5 | | **0.7*** | | 7.1 | | **0.9**** | | -- | | **8.1***** | | | 18.2% | |  |
| Pensacola | -3.8 | **7.0***** | | | | **0.7***** | | -1.5 | | 0.8 | | -- | | -- | | -- | -- | | **-1.7***** | | -- | | -- | | **0.4**** | | -- | | | 29.1% | |  |
| Apalachicola | -- | 4.4 | | | | 0.3 | | -1.1 | | NA | | -- | | -- | | -- | NA | | -- | | NA | | -1.5 | | NA | | NA | | | 7.9% | |  |
|  |  |  | | | |  | |  | |  | |  | |  | |  |  | |  | |  | | Average Variance | | | | | | | 17.91% | |  |
