# Supplementary material for: Evaluation of Blue Crab, Callinectes sapidus, Megalopal Settlement and Condition during the Deepwater Horizon Oil Spill
Source: PLoS One. 2015 Aug 13;10(8):e0135791. doi: 10.1371/journal.pone.0135791 (PMC4535880; doi:10.1371/journal.pone.0135791)

**Supplement 4 - Seasonal and lunar trends in hydrodynamics and wind variables**

To explore the potential for seasonal and lunar trends in the independent variables (daily sea level flux, maximum sea level height, night sea level height, and the N-S wind component), we first fit seasonal and lunar trends as described in the Methods section. For each variable, we give the AIC selection table for both the Seasonal and the Lunar models. We also plot the time series data for each variable and the best fitted trend. Blue indicates year 2010 data and trend, orange indicates year 2011 data and trend, and black indicates both 2010 and 2011 trend. In general, seasonal trends were apparent and usually slightly different across years and sites, but they were never very pronounced. As expected, lunar trends were more pronounced in the hydrodynamic variables (daily sea level flux, daily maximum sea level, mean night sea level) and varied slightly across the two years. These lunar trends were somewhat similar to the lunar trend found in the Blue Crab settlement data, so we undertook a series of alternate analyses to determine whether this was confounding our interpretation of hydrodynamic and wind effects in settlement.

**Daily Sea Level Flux**

| **Seasonal Model** | k | AIC | ∆AIC | **Lunar Model** | k | AIC | ∆AIC |
| --- | --- | --- | --- | --- | --- | --- | --- |
| **Site** | 16 | -1780 | 0 | **none** | 5 | -1896 | 0 |
| Year*Site | 28 | -1766 | 13.9 | **Year** | 9 | -1895 | 0.61 |
| Year | 7 | -1542 | 237.2 | Site | 21 | -1876 | 19.3 |
| none | 4 | -1527 | 368.7 | Site*Year | 37 | -1852 | 43.39 |
| null | 2 | -1500 | 395.9 | null | 2 | -1808 | 88.07 |


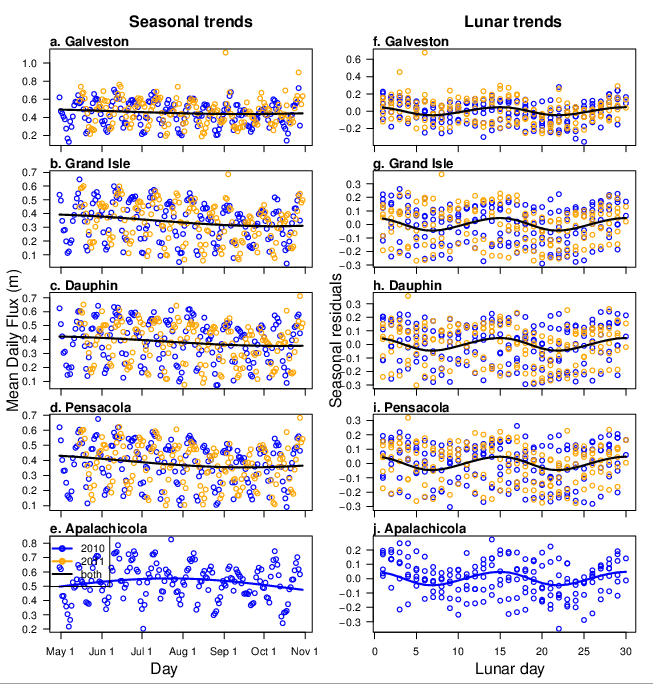


**Max Daily Sea Level**

| **Seasonal Model** | k | AIC | ∆AIC | **Lunar Model** | k | AIC | ∆AIC |
| --- | --- | --- | --- | --- | --- | --- | --- |
| **Year*Site** | 28 | -2226.7 | 0 | **Year** | 9 | -2301.6 | 0 |
| Year | 7 | -2202.4 | 24.301 | none | 5 | -2289.2 | 12.4 |
| Site | 16 | -2065.7 | 161.013 | null | 2 | -2278.7 | 22.9 |
| none | 4 | -2017.4 | 284.219 | Site | 21 | -2278.0 | 23.6 |
| null | 2 | -1991.8 | 309.755 | Year*Site | 37 | -2265.3 | 36.3 |


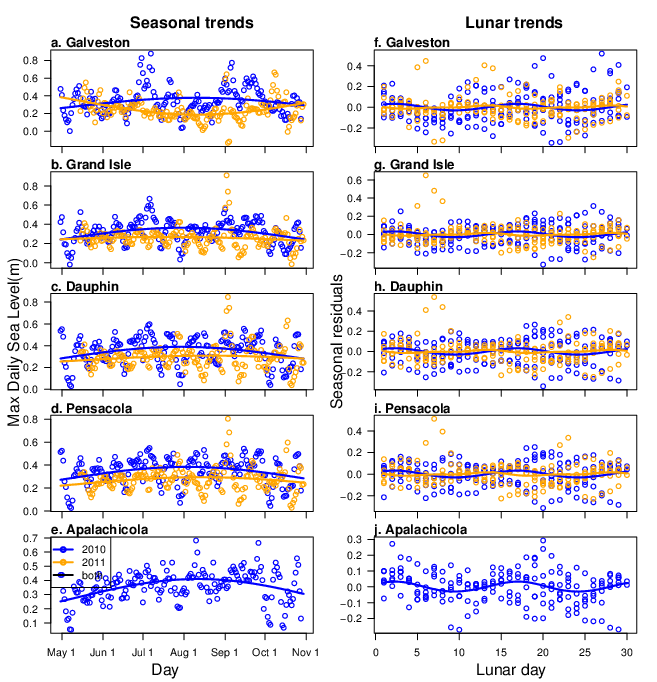


**Mean Night Sea Level**

| **Seasonal Model** | k | AIC | ∆AIC | **Lunar Model** | k | AIC | ∆AIC |
| --- | --- | --- | --- | --- | --- | --- | --- |
| **Year** | 7 | -1904.0 | 0 | **Year** | 9 | -2175.7 | 0 |
| Year*Site | 28 | -1901.7 | 2.3 | none | 5 | -2133.4 | 42.4 |
| none | 4 | -1855.3 | 48.7 | Year*Site | 37 | -2130.6 | 45.1 |
| Site | 16 | -1843.9 | 60.1 | Site | 21 | -2115.1 | 60.6 |
| null | 2 | -1809.2 | 94.8 | null | 2 | -1953.7 | 222.1 |


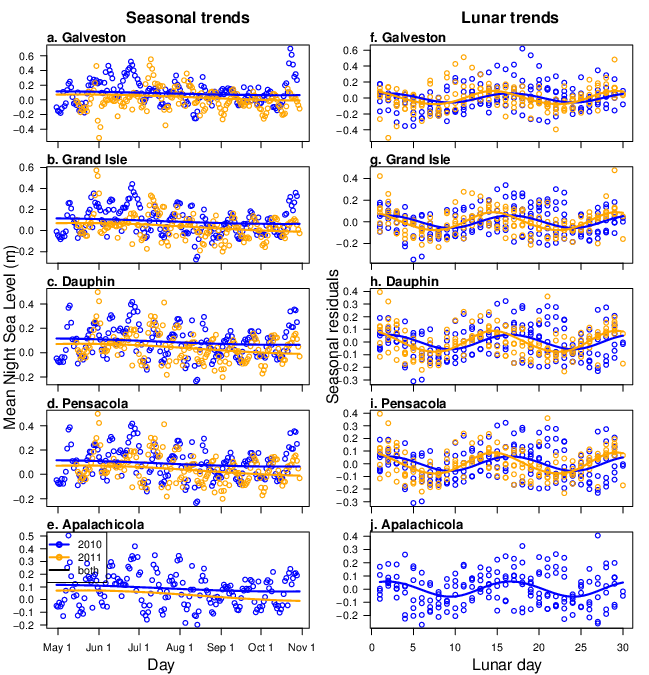


**N-S Wind Component**

| Seasonal Model | k | AIC | ∆AIC | Lunar Model | k | AIC | ∆AIC |
| --- | --- | --- | --- | --- | --- | --- | --- |
| **Year*Site** | 28 | 7183.1 | 0 | **Year** | 9 | 7117.0 | 0 |
| Site | 16 | 7216.3 | 33.2 | none | 5 | 7128.2 | 11.2 |
| Year | 7 | 7496.6 | 313.5 | null | 2 | 7131.1 | 14.1 |
| none | 4 | 7506.3 | 323.2 | Year*Site | 37 | 7135.2 | 18.2 |
| null | 2 | 7677.6 | 494.5 | Site | 21 | 7135.2 | 18.2 |


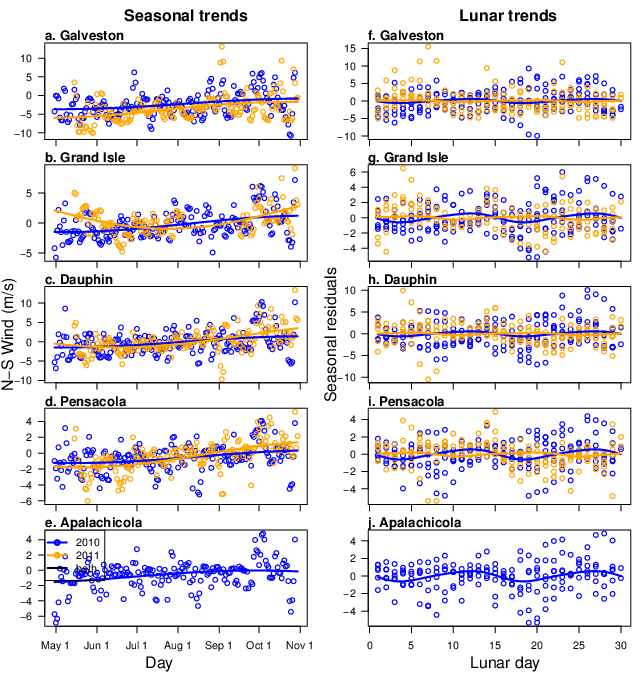

Supplement: S1 File — (DOCX) [file pone.0135791.s006.docx]
