# Supplementary material for: Evaluation of Blue Crab, Callinectes sapidus, Megalopal Settlement and Condition during the Deepwater Horizon Oil Spill
Source: PLoS One. 2015 Aug 13;10(8):e0135791. doi: 10.1371/journal.pone.0135791 (PMC4535880; doi:10.1371/journal.pone.0135791)

**Supplement 6 - Time Series Plots of *Callinectes sp.* Settlement Rates, Hydrodynamic and Wind Variables**

Mean daily *Callinectes spp*. settlement rates along with hydrodynamic and wind variables. Settlement rates are normalized by the maximum settlement rate at each site and year and plotted as solid black bars. See Appendix 1 for plots of raw (unnormalized) settlement data. Daily Flux and Daily Max Sea Level were both scaled by subtracting the minimum value for that site and year, Alongshore wind was divided by 5, and NS and EW wind components were divided by 10. This scaling was done to improve readability of the plots. Mean Night Sea Level was not scaled. New moon and full moon are represented by empty and filled circles respectively. In the hydrodynamics plots “Flux” = daily sea level flux in meters, “Max” is daily maximum sea level and “Night” is the mean nightly sea level. In the wind plots, “Along” is the alongshore wind component, “N-S” is the North-South or *u* wind component, and “E-W” is the East-West or *v* component. The x-axis in all plots the Julian day.


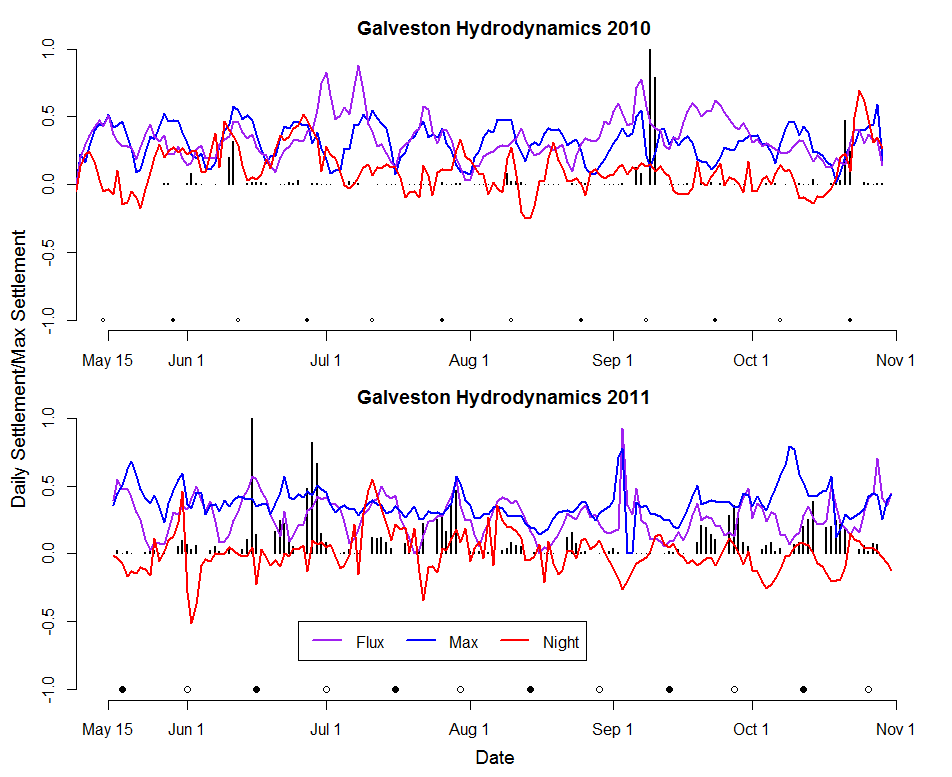


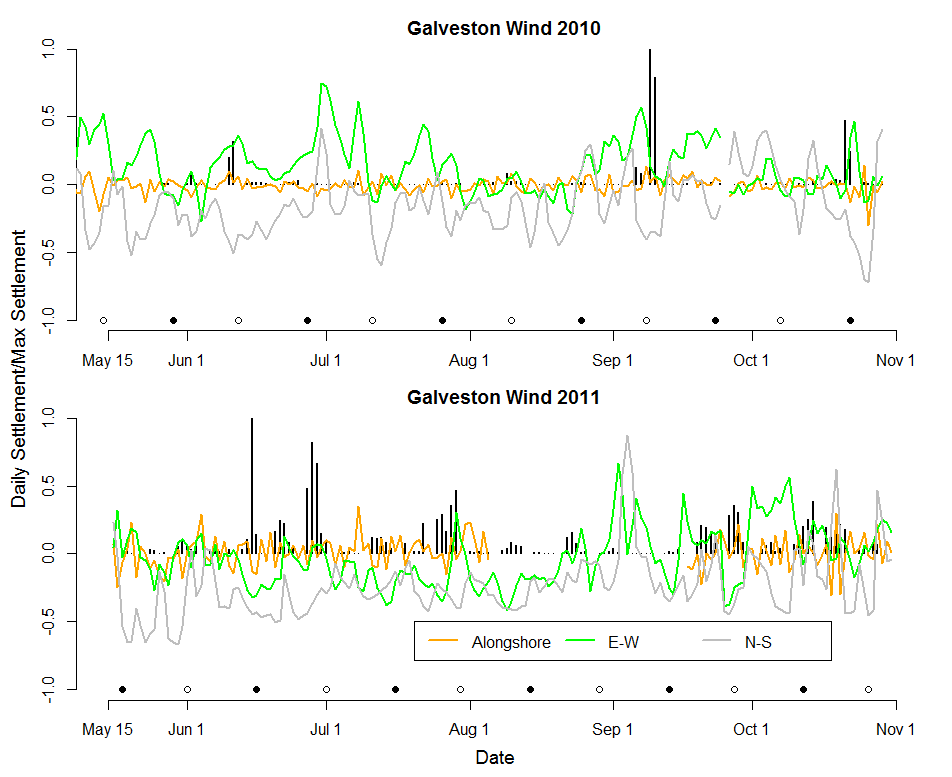


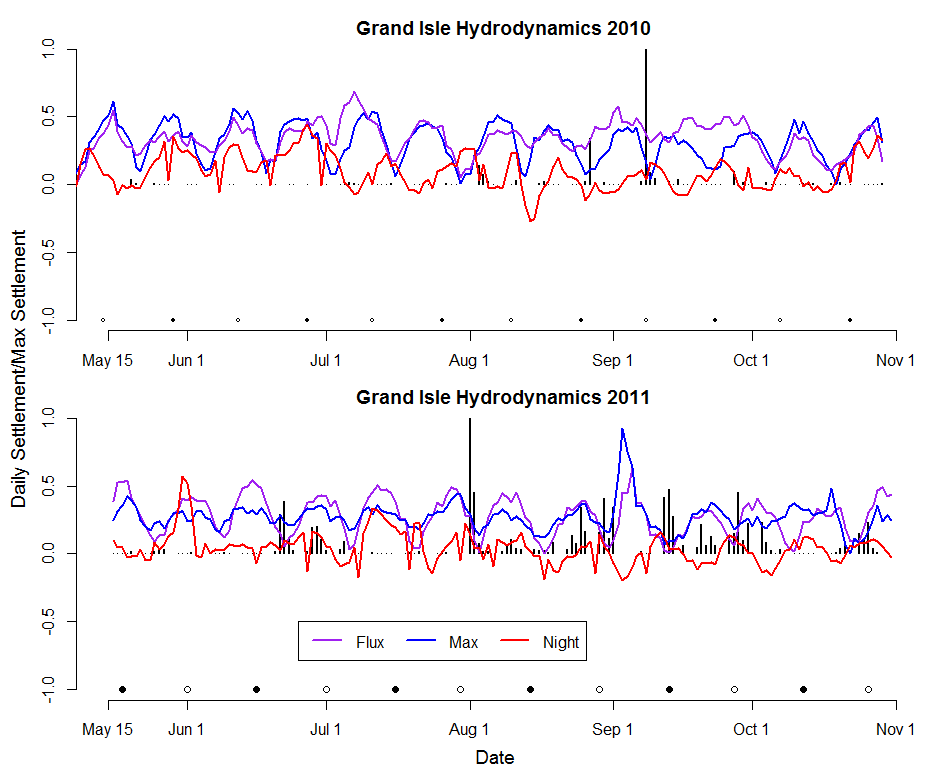


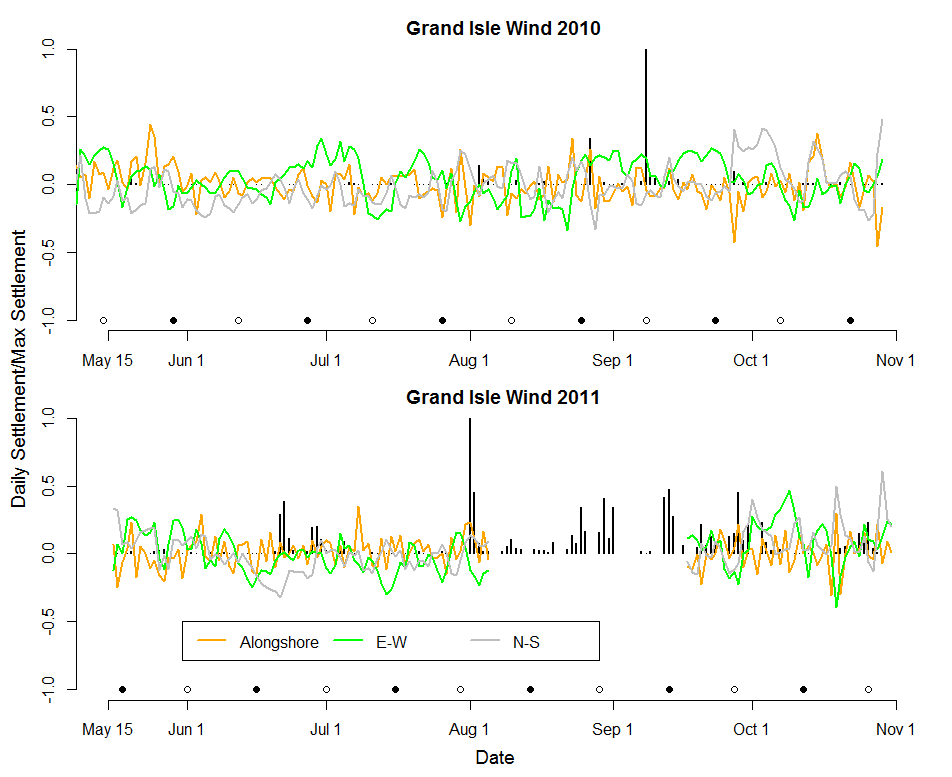


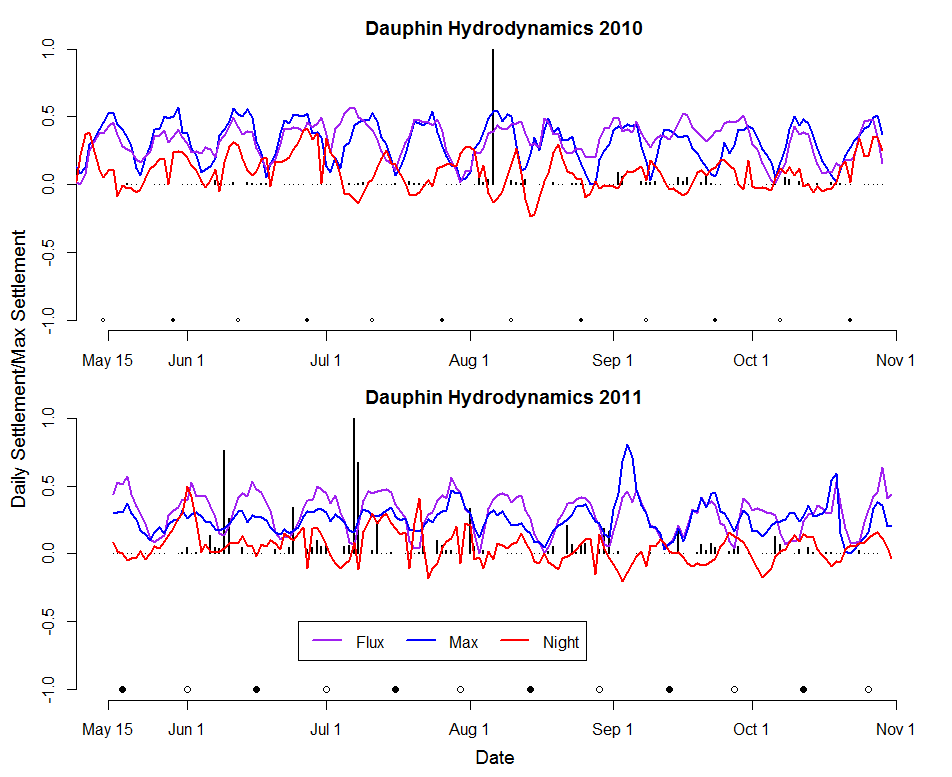


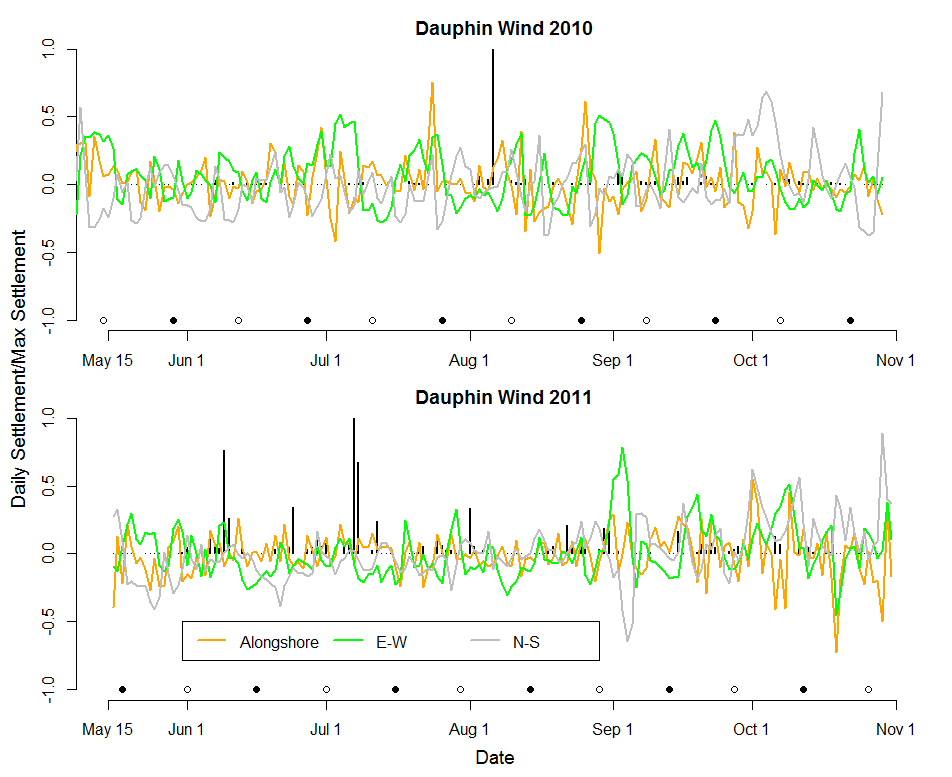


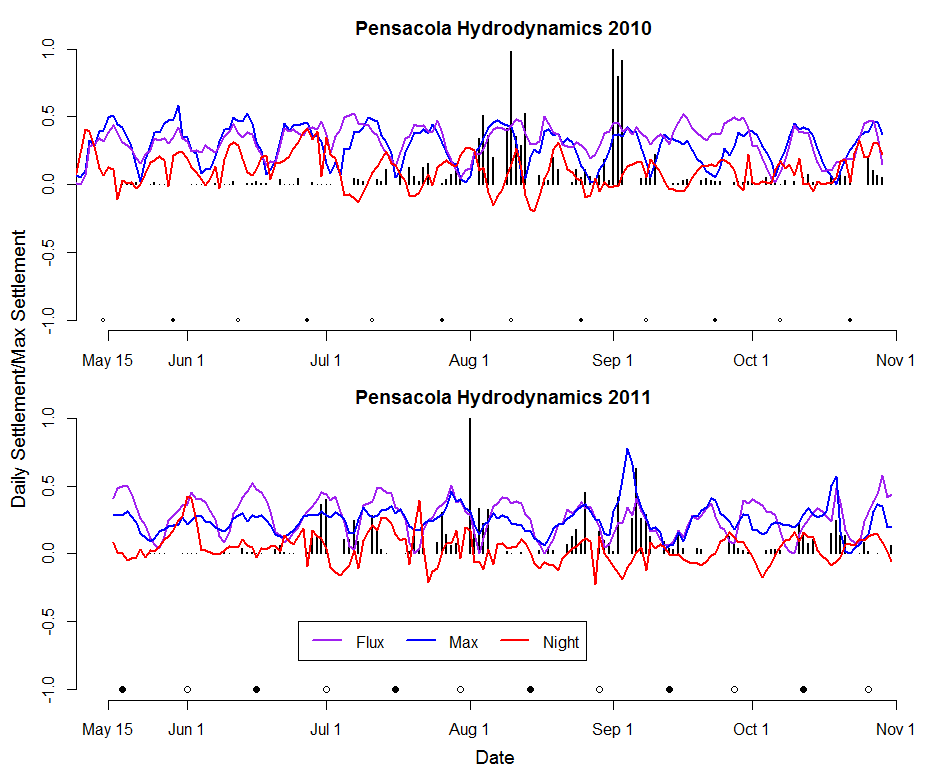


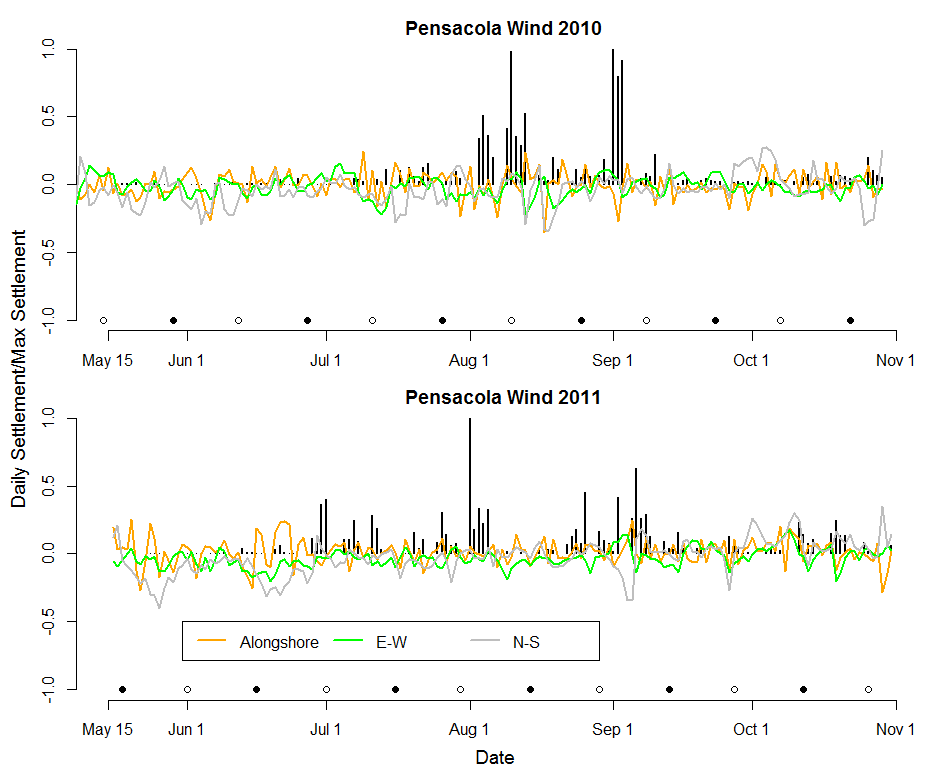


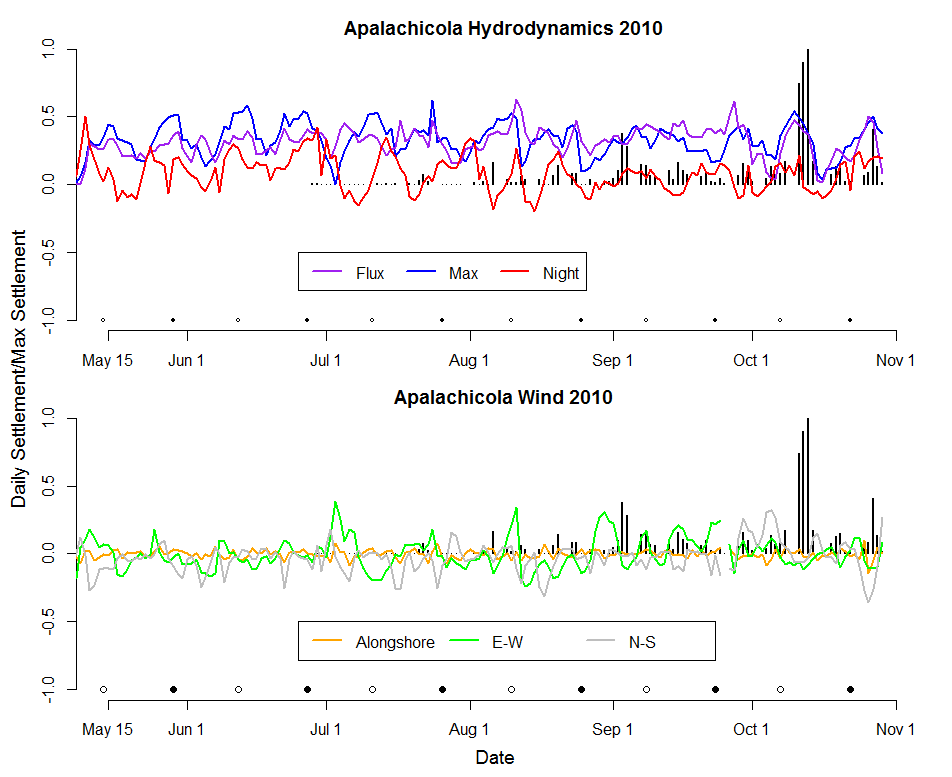

Supplement: S2 File — (DOCX) [file pone.0135791.s007.docx]
